# Emergency call training in primary school children: effects of instruction format and dispatcher influence

**DOI:** 10.3389/fped.2026.1782304

**Published:** 2026-04-22

**Authors:** Anthea Peters, Soyhan Bagci, Nicole Müller

**Affiliations:** 1 Department of Paediatrics, University Hospital Bonn, Bonn, Germany; 2 Department of Paediatric Cardiology, University Hospital Bonn, Bonn, Germany

**Keywords:** CPR, emergency call, firstaid education, primary school children, simulation-based training

## Abstract

**Background:**

Early activation of emergency medical services is the first link in the chain of survival, yet training in emergency call competence has received limited attention compared to cardiopulmonary resuscitation (CPR). Teaching children to place an emergency call may provide a crucial and age-appropriate entry point for first aid education.

**Methods:**

In this prospective randomized, controlled experimental study, 71 pupils (7–9 years old) from second and third grades of a German elementary school were randomized to frontal instruction or simulation-based training; a control group completed a test call prior to training. Test calls were conducted using mobile phones, assessed by trained dispatchers, and scored with a validated evaluation sheet (maximum 17 points; ≥11 defined as sufficient).

**Results:**

Overall, 84.5% of children achieved a sufficient emergency call. After training, 100% recalled the correct emergency number, compared with 78% in the control group (*p* = 0.042). No significant differences were found between frontal and simulation groups. Grade level was a strong predictor: 91% of third graders delivered sufficient calls, compared to 64% of second graders. Notably, 9.9% of all children were unable to speak after the dispatcher's greeting, and 12.5% could not provide a location, the majority being second graders. Dispatcher behavior strongly influenced outcomes, with significant differences in 11 of 13 assessed items.

**Conclusion:**

Primary school children are capable of placing sufficient emergency calls after a brief training. Instruction format (frontal vs. simulation) had little impact, suggesting that emergency call teaching can be delivered efficiently in classroom settings, preserving simulation time for psychomotor CPR skills. Future trainings should emphasize reducing fear of speaking, ensuring knowledge of addresses, and incorporating dispatcher training to optimize communication with child callers.

## 1 Introduction

Each year, more than 50,000 individuals in Germany and approximately 300,000 across Europe experience an out-of-hospital cardiac arrest (OHCA), making it the third-leading cause of death in industrialized nations (1). Despite decades of advancement, the survival rate to hospital discharge remains low—only 8%–10% (2, 3). Given that irreversible neuronal damage occurs within 3–5 minutes after circulatory arrest and that emergency medical services (EMS) typically require 5–8 minutes to arrive (4–6), immediate layperson cardiopulmonary resuscitation (CPR) is crucial. Data from the EuReCa TWO study demonstrated that survival rates more than doubled from 4.3% to 9.1% when bystander CPR was performed (7). Estimates suggest that up to 200,000 additional lives could be saved annually in the United States and Europe if all witnessed arrests elicited a layperson response (8, 9).

In this context, training schoolchildren in basic life support (BLS) has emerged as an increasingly popular public-health strategy across Europe. Integrating CPR instruction into the school curriculum ensures broad population coverage, particularly among socioeconomically disadvantaged groups-those at higher risk for sudden cardiac death and less likely to receive bystander intervention (10, 11). Children also serve as powerful multipliers: initiatives like the “Kids Save Lives” campaign of the European Resuscitation Council (ERC) encourage each trained child to instruct 10 others in their environment (12). Studies confirm that distributing training materials to schoolchildren enables indirect training of 1.7 to 2.5 additional individuals per child—most effectively when led by girls (13–15).

In Denmark, nationwide CPR initiatives, including mandatory school-based training, were associated with marked increases in bystander CPR rates and improved survival (16). Such findings demonstrate not only an increased willingness to act but also a measurable improvements in patient outcomes.

Despite this progress, the first link in the chain of survival—initiating the emergency call—has received disproportionately little attention in both training and research. The ERC recommends that children at a minimum be taught the correct emergency number (9), but there is little consensus on the best pedagogical method, particularly for younger age groups. Timely activation of EMS is paramount, as bystander CPR merely bridges the gap until professional help arrives. Survival was 0% when a relative was called first vs. 11% with immediate EMS activation in a US OHCA study (22).

The standard age to begin CPR training is grade 7, based on the physical strength required to perform effective chest compressions (17–19). However, placing an emergency call requires no such physical abilities, suggesting the potential for much earlier skill acquisition.

Evidence suggests that even young children (4–7 years) can acquire the ability to place an emergency call following structured instruction (20, 21). In older children, this skill appears particularly robust: among Italian children aged 11–12, the emergency call was the most reliably performed task in simulated scenarios, with 70% initiating it spontaneously and a further 20% after minimal prompting (25). Similarly, studies in primary school children show substantial improvements after training (20, 26), with 77% recalling the emergency number and 50% providing correct information compared to 16% and 6% in untrained controls (27). As dispatchers can guide callers through recognition of cardiac arrest and CPR (telephone-assisted CPR, T-CPR), early activation of emergency medical services should be considered a fundamental goal of life-support education (23, 24). However, not all educational approaches are equally effective. The “Save City” program, evaluated in 5–6-year-olds in Ohio, found no significant performance difference between intervention and control groups, highlighting the importance of instructional design, duration, and group dynamics (28).

This prospective, randomized, controlled experimental study therefore addresses two central questions:

1. Can primary school children acquire both cognitive knowledge and practical competence required to place an effective emergency call following a short training session?
2. Is simulation-based instruction superior to traditional frontal teaching for this purpose?

To address these questions, 71 children from 2nd and 3rd grade classes at a German elementary school participated in a structured first-aid course. One half received simulation-based training, while the other half received frontal teaching; A control group completed a simulated emergency call prior to training to establish baseline comparison.

## 2 Material and methods

### 2.1 Participants

71 second- and third-grade pupils (aged 7–9 years) from a public elementary school in Bad Honnef, Germany, were recruited. The four participating classes were randomly assigned to either frontal teaching or simulation-based instruction.

Within each class, pupils were further randomized to a control (simulated emergency call conducted before training) or intervention group (test call conducted after training) using a simple lottery procedure, in which children drew numbered lots from a container to determine group allocation. Written informed consent was obtained from both children and their legal guardians.

### 2.2 Conception and implementation of the teaching units

In the frontal teaching group, pupils were expected to state the correct emergency number and provide the dispatcher-requested details. In the simulation group, pupils were expected to state the emergency number and complete a full simulated emergency call.

The entire first aid course lasted 90 minutes, with the emergency call module comprising approximately 10 minutes, plus an additional 10 minutes of role play in the simulation group. A senior physician from the pediatric emergency department delivered the course.

### 2.3 Conception of the test emergency call

Two evaluators served as dispatchers: a nurse with prior experience as an emergency dispatcher and a paramedic. Both were specifically trained for their roles and conducted test emergency calls according to a standardized written protocol. During each call, a second trained assessor was present and monitored the conversation via loudspeaker.

Prior to testing, pupils received a standardized pre-briefing, informing them that they would be making a simulated emergency call. It was emphasized that the emergency dispatch center had been notified of the study and that no real ambulance would be dispatched. The briefing was intended to prevent anxiety about false alarms and to discourage any unsupervised “test calls” outside the study setting.

**Figure 1:**
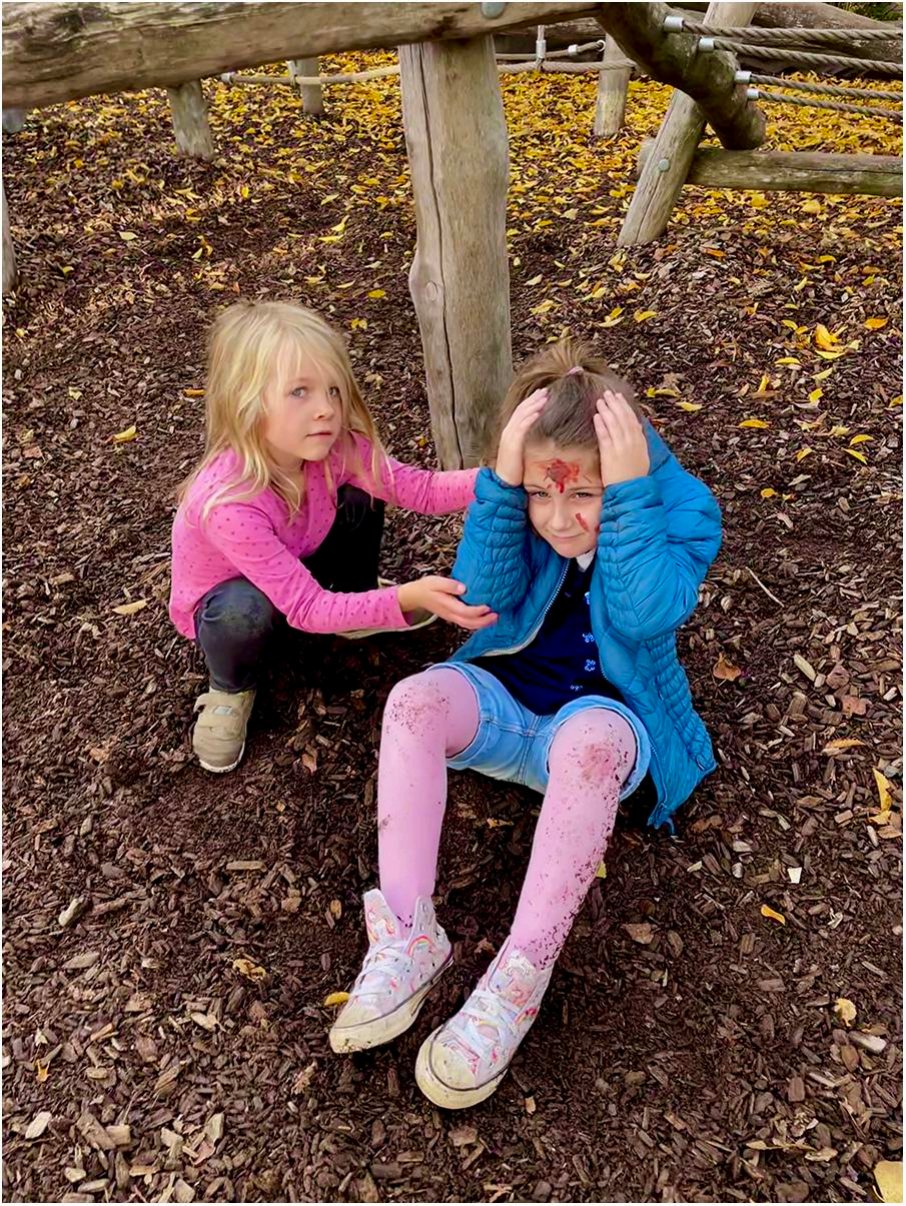
Image used for the simulated emergency call.

Emergency scenarios were presented using photographic images rather than live simulations to ensure psychological safety. Images depicted familiar playground accidents and were taken on the school premises, enabling pupils to correctly identify the location. Photographs were alternated throughout the day to avoid information transfer between pupils. The depicted child's open eyes indicated preserved consciousness (Figure 1).

Each test began with the following instruction: “*On your way home from school, you see the following situation on the playground (picture shown). You want to make an emergency call. Can you tell me the number?*” After stating the number, pupils were then instructed: “Thank you. Now you can place the emergency call.”

**Figure 2:**
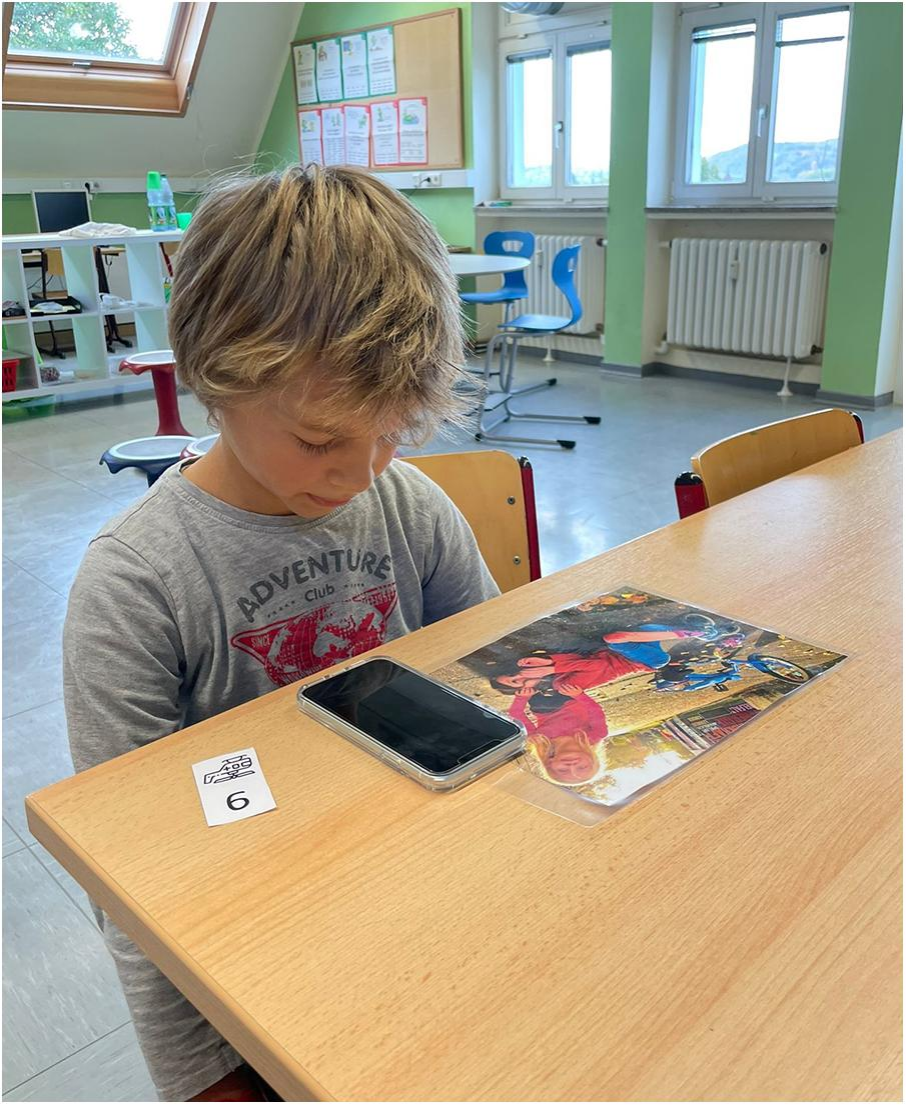
Pupil during the simulated emergency call.

Calls were performed using a mobile phone with the number “112” pre-programmed under the corresponding contact name. Upon connection, the child heard the standardized dispatcher greeting: “*Emergency Dispatch Center Bonn, where is the emergency?*” Pupils could choose either to use the loudspeaker function or hold the phone to their ear (Figure 2).

Following each call, the dispatcher and the second assessor jointly assigned a performance score using the predefined standardized evaluation form.

### 2.4 Evaluation sheets

Emergency calls were scored using a structured predefined standardized evaluation form that covered both content and communication aspects. Items were weighted according to their relative importance. The maximum score was 17 points and a total of ≥11 points defined a sufficient call. The form was peer-reviewed by emergency physicians, paramedics, and teachers. Evaluators who received the calls were trained in applying the form. Scores were assigned jointly by the dispatcher and second assessor after each call by consensus; therefore, no formal inter-rater reliability coefficient was calculated.

### 2.5 Statistical evaluation

Data were analyzed using IBM SPSS Statistics (Version 26, IBM Corp., Armonk, NY, USA). Descriptive statistics and mean comparisons were performed, with outliers retained to represent children who were unable to complete an emergency call. Homogeneity of variances was tested with Levene's test; t-tests or Welch's tests were applied as appropriate, with statistical significance set at *p* < 0.05. A multifactorial analysis of variance (ANOVA) was used to assess the effects of grade level and training modality, and group composition was examined using chi-square tests.

## 3 Results

### 3.1 Descriptive analysis

Of the 84 pupils invited to participate, parental consent was obtained for 71, who were therefore included in the study. Table 1 presents the distribution of pupils across the study groups. No significant difference was found in group distribution across classes (*p* = 0.321).

**Table 1:** Distribution of pupils into groups by class, cross-table.

Group		2nd grade	3rd grade	Total
Control group	*n* (%)	9 (50,0%)	9 (50,0%)	18 (100%)
Frontal teaching group	*n* (%)	9 (39,1%)	14 (60,9%)	23 (100%)
Simulation group	*n* (%)	18 (60,0%)	12 (40,0%)	30 (100%)
Total	*n* (%)	36 (50,7%)	35 (49,3%)	71 (100%)

### 3.2 Distribution of total scores

Table 2 summarizes the distribution of total scores. Overall, 60 out of 71 pupils (84.5%) achieved more than 11 points, thereby demonstrating a sufficient emergency call performance.

**Table 2:** Distribution of total scores across groups.

Number of points	Control group *n* (%)	Frontal teaching group *n* (%)	Simulation group *n* (%)	Total *n* (%)
<4 points	2 (11,1%)	2 (8,7%)	4 (13,3%)	8 (11,2%)
4–10 points	2 (11,1%)	1 (4,3%)	0 (0,0%)	3 (4,2%)
11–14 points	5 (27,8%)	17 (73,9%)	22 (73,3%)	44 (62,0%)
>14 points	9 (50,0%)	3 (13,0%)	4 (13,3%)	16 (22,5%)
**Total**	**18** (**100%)**	**23** (**100%)**	**30** (**100%)**	**71** (**100%)**

Bold values indicate total number of participants per group (*n*).

### 3.3 Effects of first aid training

No significant difference was observed between control and trained pupils regarding total scores (Table 3). A significant improvement was found only in knowledge of the emergency number (*p* = 0.042), with all 53 trained pupils correctly recalling the number after the intervention.

**Table 3:** Results for test calls before and after training.

Test-item	Control group (*n* = 18) mean [±SD]	Trained (*n* = 53) mean[±SD]	*p*-value
Emergency number	2,33 [±1,28]	3 [±0]	0,042*
Total score	12,19 [±4,53]	12,26 [±3,47]	0,946^ns^

Results in average values with specification of standard deviation (SD), ns, not significant.

* Significant.

No significant differences were identified between frontal-teaching and simulation-training groups.

### 3.4 Comparison by grade level

When comparing grades irrespective of training modality (Table 4), third-grade pupils consistently outperformed second-grade pupils, with significant differences in several items, including correct emergency number, name, location, and type of emergency.

**Table 4:** Comparison of all participants in the 2nd and 3rd grade.

Test-item	2nd grade (*n* = 36) mean [±SD]	3rd grade (*n* = 37) mean [±SD]	*p*-value
Emergency number	2,67 [±0,96]	3 [±0]	0,044*
Speaks	1,56 [±0,69]	1,83 [±0,57]	0,074^ns^
Slowly	0,86 [±0,35]	0,9 [±0,29]	0,613^ns^
Loudly	0,85 [±0,35]	0,91 [±0,28]	0,383^ns^
Clearly	0,85 [±0,35]	0,9 [±0,29]	0,497^ns^
Awaits Questions	1,67 [±0,76]	1,83 [±0,57]	0,311^ns^
States name	0,46 [±0,18]	0,61 [±0,30]	0,011*
Location (“Where?”)	0,38 [±0,28]	0,59 [±0,28]	0,002**
Situation (“What?”)	0,32 [±0,30]	0,6 [±0,32]	*p* < 0,001***
Person (“Who?”)	0,5 [±0,27]	0,63 [±0,31]	0,064^ns^
Number (“How many?”)	0,46 [±0,22]	0,63 [±0,31]	0,009**
Injury location	0,5 [±0,29]	0,64 [±0,31]	0,05^ns^
Unconsciousness	0,42 [±0,19]	0,44 [±0,16]	0,533^ns^
Total score	11,35 [±4,01]	13,17 [±3,21]	0,038^ns^

Results in mean values with indication of the SD, ns, not significant.

* Significant.

** Very significant.

*** Highly significant.

In Grade 2 (Table 5), trained pupils performed better than controls in most items, with a significant improvement in recalling the emergency number.

**Table 5:** Control group and trained participants from 2nd grade.

	2nd Grade		
Test-item	Control group (*n* = 9) mean [±SD]	Trained (*n* = 27) mean [±SD]	*p*-value
Emergency number	1,67 [±1,58]	3 [±0]	0,035*
Speaks	1,44 [±0,73]	1,59 [±0,69]	0,587^ns^
Slowly	0,78 [±0,44]	0,89 [±0,32]	0,418^ns^
Loudly	0,72 [±0,44]	0,89 [±0,32]	0,228^ns^
Clearly	0,72 [±0,44]	0,89 [±0,32]	0,228^ns^
Awaits Questions	1,33 [±1]	1,78 [±0,64]	0,239^ns^
States name	0,5 [±0,25]	0,44 [±0,16]	0,441^ns^
Location (“Where?”)	0,44 [±0,30]	0,35 [±0,27]	0,393^ns^
Situation (“What?”)	0,39 [±0,33]	0,30 [±0,29]	0,425^ns^
Person (“Who?”)	0,5 [±0,35]	0,5 [±0,24]	1^ns^
Number (“How many?”)	0,5 [±0,35]	0,44 [±0,16]	0,519^ns^
Injury location	0,39 [±0,22]	0,54 [±0,31]	0,193^ns^
Unconsciousness	0,39 [±0,22]	0,43 [±0,18]	0,618^ns^
Total score	9,56 [±5,23]	11,94 [±3,43]	0,123^ns^

Results as mean values with indication of standard deviation (SD), ns, not significant.

* Significant.

In Grade 3 (Table 6), control pupils outperformed trained pupils in several content-related items (e.g., location, type of emergency, number of persons involved) as well as in total scores (*p* = 0.005). No significant differences were observed in communication-related items.

**Table 6:** Control group and trained participants of 3rd grade.

	3rd grade		
Test-item	Control group (*n* = 9) mean [±SD]	Trained (*n* = 27) mean [±SD]	*p*-value
Emergency number	3 [±0]	3 [±0]	
Speaks	2 [±0]	1,77 [±0,65]	0,083^ns^
Slowly	0,94 [±0,17]	0,88 [±0,33]	0,604^ns^
Loudly	1 [±0]	0,88 [±0,33]	0,083^ns^
Clearly	0,94 [±0,17]	0,88 [±0,33]	0,604^ns^
Awaits Questions	2 [±0]	1,77 [±0,65]	0,083^ns^
States name	1 [±0]	0,48 [±0,22]	p < 0,001***
Location (“Where?”)	0,89 [±0,33]	0,48 [±0,17]	0,006**
Situation (“What?”)	1 [±0]	0,46 [±0,24]	p < 0,001***
Person (“Who?”)	1 [±0]	0,5 [±0,24]	p < 0,001***
Number (“How many?”)	1 [±0]	0,5 [±0,24]	p < 0,001***
Injury location	0,89 [±0,22]	0,56 [±0,29]	0,004**
Unconsciousness	0,5 [±0]	0,42 [±0,18]	0,043**
Total score	14,83 [±0,75]	12,6 [±3,54]	0,005**

Results as mean values with indication of the standard deviation (SD), ns, not significant.

** Very significant.

*** Highly significant.

### 3.5 Multifactorial analysis of variance (ANOVA)

A multifactorial ANOVA was conducted to assess the effects of group (control, frontal teaching, simulation), grade level (2nd vs. 3rd grade), and their interaction on total scores (Table 7).

**Table 7:** Results of the multifactorial analysis of variance (ANOVA) examining the effects of group (control, frontal instruction, simulation instruction), grade level (second vs. third grade), and their interaction on total emergency call score.

	df	Mean square	F	Sig.
Corrected Model^a^	5	28,696	2,246	0,06
Group	2	0,298	0,023	0,977
Grade	1	83.562	6,54	0,013
Group x Grade	2	42,262	3,3	0,043
Error	65			
Total	71			

df, degrees of freedom.

^a^ R-squared = 0.147 (adjusted R-squared = 0.082).

The overall model showed no statistical significance [F(5,65) = 2.246, *p* = 0.060]. Group membership alone was not significantly associated with total score [F(2,65) = 0.023, *p* = 0.977]. Grade level showed a significant main effect [F(1,65) = 6.540, *p* = 0.013], and there was a significant interaction between grade level and group assignment [F(2,65) = 3.300, *p* = 0.043].

The lowest mean score was observed in the second-grade control group (M = 9.56), while the highest was in the third-grade control group (M = 14.83). Among second-grade pupils, simulation training produced slightly higher mean scores (M = 12.19) than frontal instruction (M = 11.44), although the difference was not significant. In third grade, the pattern reversed, with the frontal-instruction group outperforming the simulation group (M = 13.14 vs. 11.95), also without statistical significance. Notably, second-grade pupils in the simulation group achieved mean scores comparable to those of third-grade pupils, thereby narrowing the pre-existing performance gap (Table 8).

**Table 8:** Mean of total scores in comparison between grades and individual groups.

Group	2nd grade	3rd grade	total
Control group	9,56	14,83	12,19
Frontal teaching group	11,44	13,14	12,10
Simulation group	12,19	11,95	12,48
Total score	11,35	13,17	12,25

### 3.6 Effects of the dispatcher

**Figure 3:**
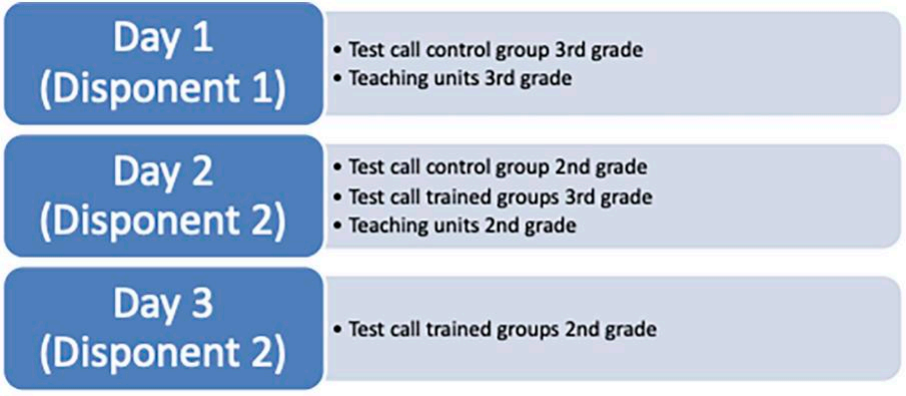
Distribution of groups and dispatchers over the different test days.

The assessments were conducted over three consecutive days by two different dispatchers (see Figure 3). Analysis revealed that pupils in the third-grade control group achieved particularly high scores when tested by Dispatcher 1.

Table 9 presents *p*-values for comparisons of individual items by dispatcher. Significant differences between the two dispatchers were observed for 11 of 13 items, including the overall total score.

**Table 9:** Item comparison by dispatcher.

Item	*p*-Value
Speaks	<0.001
Slowly	0.526
Loudly	0.002
Clearly	0.261
Awaits Questions	0.002
States name	<0.001
Location (“Where?”)	<0.001
Situation (“What?”)	<0.001
Person (“Who?”)	<0.001
Number (“How many?”)	<0.001
Injury location	<0.001
Unconsciousness	0.001
Total score	<0.001

**Figure 4:**
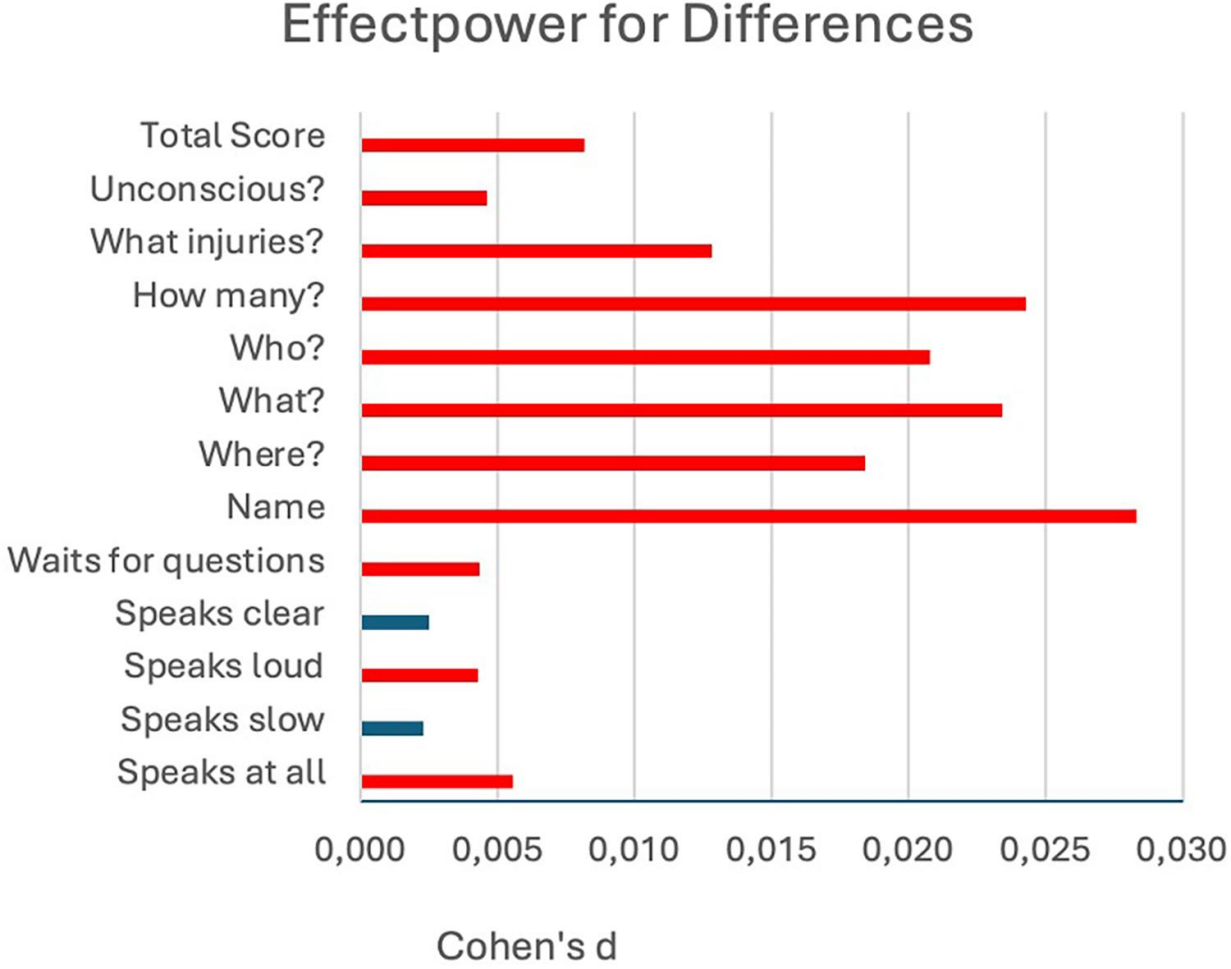
Cohen's d for the individual items. Significant differences are shown in red.

Figure 4 illustrates the effect sizes (Cohen's d) for each item, with significant differences highlighted in red.

## 4 Discussion

This study is the first to examine the comparative effectiveness of different didactic formats for teaching primary school children how to place an emergency call. Early activation of emergency medical services is widely recognized as the first link in the chain of survival, yet training in emergency call competence has received comparatively little attention in school-based first aid education. Our results confirm that even elementary school–aged pupils are capable of performing a sufficient emergency call, underscoring the importance of integrating this topic into early emergency care education.

### 4.1 Effect of first aid training

The pronounced training effect was that after the intervention, all children knew the correct emergency number—knowledge that can be lifesaving in case of an emergency. In contrast, 22% of pupils in the control group (*n* = 4), all second graders, did not know the correct number. This raises the question of whether knowledge of the emergency number is age-dependent.

Grade level emerged as one of the strongest predictors of emergency-call competence. This finding is consistent with previous studies demonstrating age-dependent improvements in cognitive and communicative abilities relevant to emergency response. While 91% of third-grade pupils delivered a sufficient call, only 64% of second graders did. Superior familiarity with home address and the emergency number among older pupils essentially explained this difference. Comparable age effects have been reported previously: Huber et al. (16) found similar age-related differences in recognizing emergencies and placing calls, although only 20% of 2nd–3rd graders in their sample knew the emergency number. In a UK study of 10–12-year-olds, 97.8% already knew the emergency number before training, increasing to 100% afterwards (17). Another UK trial comparing 13–16-year-olds trained in BLS/ AED with controls found no advantage of training, implying that above a certain age, such knowledge becomes part of general education (18). These findings suggest that knowledge of the emergency number develops with age but cannot be assumed even in older pupils, particularly considering differences in background, migration history, or special educational needs. Importantly, none of the cited studies showed that 100% of pupils knew the correct number prior to training, underscoring the need for systematic instruction in schools. In the present study, the control group already exhibited high baseline knowledge, limiting measurable post-training gains. Given the school's relatively affluent catchment area, larger effects could be expected in settings with lower baseline knowledge or limited prior exposure to first-aid topics.

### 4.2 Difficulties in placing emergency calls

An unexpected finding of the present study was that 7 of 71 pupils (9.9%) were unable to speak to the dispatcher after hearing the initial greeting. Some even hung up in shock, handed the phone back to the supervising person or began to cry. This result demonstrates that, despite knowing the emergency number and presumably the correct procedure, a subset of children is unable to engage in communication with the dispatcher.

Another relevant issue concerns knowledge of the location. A total of 12.5% of pupils (*n* = 8) who were able to speak to the dispatcher could not provide the ambulance's destination. Notably, seven of these children were from the second grade.

### 4.3 Influence of the dispatcher

A particularly striking and unexpected finding was the strong influence of the dispatcher on children's performance. In the present study, third-grade pupils performed significantly worse after training compared with their baseline scores (14.8 vs. 12.7 points, *p* = 0.005). This result was surprising, as a deterioration in performance after training is counterintuitive and inconsistent with all other trends observed. Upon closer examination, it became evident that this apparent decline was linked to the fact that different dispatchers conducted the pre- and post-training assessments, rather than to a true loss of skill. Subsequent analysis revealed systematic and statistically significant differences between the two dispatchers across almost all evaluated items (11 of 13, including the overall score). These discrepancies most likely reflect differences in individual communication style, tone, and approach to interacting with the child callers, despite both dispatchers having received the same standardized written script and pre-briefing.

Qualitative observations and post-study interviews with the secondary evaluator, who was present during all calls, further illuminated these differences. Dispatcher 1 was described as empathetic, patient, and playful, using a gentle tone and frequent affirmations (“That's great—can you tell me more?”), which appeared to reduce anxiety and facilitate dialogue. In contrast, Dispatcher 2 was characterized as more formal, concise, and demanding, with longer pauses and a more neutral tone, which seemed to increase hesitation and anxiety among some children. Several participants became silent or needed encouragement to continue when interacting with the more authoritative dispatcher. The observed variation highlights a critical but underexplored dimension of emergency communication: the interpersonal style of the dispatcher as a determinant of caller performance, particularly among children. Existing research on adult layperson CPR has shown that dispatcher tone and empathy significantly influence caller compliance and willingness to act, yet similar data for child callers are virtually absent. To our knowledge, this is one of the first studies to demonstrate a measurable impact of dispatcher communication style on emergency call performance in children.

From a training and policy perspective, these findings suggest that dispatcher communication training should explicitly address interactions with child callers. Standardized, child-sensitive opening phrases, use of a calm and supportive tone, and awareness of developmental differences in communication ability may substantially enhance the effectiveness of telephone-assisted emergency response. In addition, integrating dispatcher voice recordings into school training modules could help familiarize children with authentic call scenarios, reducing fear and improving preparedness for real-life emergencies.

### 4.4 Effect of training format

In the present study, no significant performance differences were observed between pupils who received frontal instruction and those who participated in a simulation-based training. This finding suggests that, for the specific task of placing an emergency call, the instructional format itself plays a minor role compared with age, baseline knowledge, or the dispatcher's interaction style. This result can be explained by the predominantly cognitive nature of the required competence. The key elements—knowing the correct emergency number, recognizing the need for help, and communicating essential information—are knowledge-based and verbally mediated skills, rather than psychomotor actions. As such, they can be effectively conveyed through simple didactic methods, such as frontal instruction, storytelling, or guided classroom discussion. In contrast, simulation-based training is particularly valuable when complex sensorimotor sequences must be learned, such as chest compressions or defibrillator use. While simulations may enhance motivation and realism, their advantages may not translate into higher test scores when the outcome measure is factual recall or verbal response. These findings support a targeted use of simulation-based learning, reserving it for practical skills while conveying cognitive components through brief, reproducible classroom instruction.

From a curricular and resource perspective, frontal instruction offers clear advantages: it requires fewer facilitators, minimal equipment, and can be easily scaled to entire school populations. The most critical competency—the knowledge of the correct emergency number—can be taught efficiently through short classroom sessions or digital modules, leaving more time for hands-on CPR practice.

It is noteworthy that all participating pupils, even in the lower grades, were familiar with mobile phone use. According to recent national surveys, approximately one-quarter of 8–9-year-olds in Germany own a mobile phone, and nearly all have regular access to a caregiver's device (Statista, 2020).This aligns with the current finding that none of the participants experienced difficulty handling the phone during testing. The growing digital literacy of children therefore represents a valuable opportunity to integrate age-appropriate digital first-aid modules into primary education.

### 4.5 Priorities for further trainings

The finding that approximately 10% of pupils were unable to speak to the dispatcher after the initial greeting highlights a key emotional and communicative barrier. Despite knowing the correct emergency number and understanding the task, a subset of children appeared overwhelmed by the situation. This indicates that knowledge-based instruction alone is insufficient; children must also be prepared emotionally and behaviorally for the stress of an emergency call.

Future training should therefore include structured desensitization and confidence-building components. Short role-play sessions or audio recordings of authentic dispatcher greetings could help children become familiar with the tone and structure of real calls, reducing anxiety and promoting verbal response. Gradual exposure and positive reinforcement—such as making the first call together with a teacher or peer—can help transform fear into confidence. In the present study, this was effectively achieved by performing a joint emergency call with a team member and providing praise or small rewards afterward to ensure a positive learning experience.

Another critical aspect concerns knowledge of location and address. Even among pupils who were able to communicate with the dispatcher, 12.5% could not specify where the ambulance should be sent, most of them in second grade. This gap is pedagogically relevant, as teachers frequently report that many early schoolchildren cannot state their home address or even the town where their school is located. Such information is essential for a successful emergency call and should therefore be explicitly included as a learning objective within first-aid curricula. Practical exercises might involve children reciting their home address aloud, identifying the school's location on a map, or practicing how to describe landmarks.

In addition, the strong impact of the dispatcher's communication style observed in this study suggests that training efforts should not focus solely on the child. Dispatchers themselves should receive specialized instruction on how to recognize and support child callers. A gentle tone, encouraging phrases, and simplified questioning strategies can markedly improve the child's ability to respond under stress. Collaborative programs between schools and emergency dispatch centers could foster this two-way learning process.

Taken together, these findings emphasize that successful emergency-call education must integrate cognitive, emotional, and communicative dimensions. Beyond teaching the number “112,” children need opportunities to practice verbal interaction, stress coping, and spatial orientation. Only through such multidimensional training we can ensure that children not only know what to do—but also feel capable and confident enough to do it.

### 4.6 Limitations of study

Several limitations should be acknowledged when interpreting these findings.

First, this was a single-center study conducted in one elementary school located in a socioeconomically privileged area. The pupils’ relatively high baseline knowledge may limit generalizability to populations with lower baseline exposure to first-aid education or differing socioeconomic and cultural backgrounds. Second, the sample size was moderate (*n* = 71), and subgroup analyses by grade level and training modality further reduced statistical power, increasing the risk of type II error. As a result, smaller differences—particularly between frontal and simulation-based instruction—may not have been detected. In addition, the study was not powered *a priori* to detect small effect sizes, and therefore non-significant findings should be interpreted with caution. Larger, multicenter trials are needed to confirm the observed patterns. Third, the simulated emergency calls were conducted under controlled and low-stress conditions. While this ensured psychological safety, it may not fully replicate the emotional and environmental stressors of a real emergency. Actual behavior in genuine emergencies could therefore differ from the performance observed in this study. Fourth, two different dispatchers conducted the test calls. Although both followed a standardized script, significant differences in interaction style were observed, which may have influenced outcomes. This highlights both a methodological limitation and an important result in itself regarding dispatcher influence. Fifth, the scoring system, while peer-reviewed and standardized, remains partly subjective and may not capture all qualitative aspects of child-dispatcher communication. Lastly, the study evaluated immediate post-training performance only; long-term retention and transfer of skills were not assessed. Future studies should include follow-up assessments to determine the persistence of training effects over time.

## 5 Conclusion

Despite these limitations, the study provides novel insights into the cognitive and communicative competencies of primary school children in emergencies. It offers valuable implications for both school curricula and dispatcher training.

The dispatcher's communication style emerged as a major determinant of success, suggesting that targeted dispatcher training could complement school-based first-aid programs at minimal cost.

Children in early primary school are capable of making effective emergency calls. As the key competence is cognitive recall of the emergency number, it can be taught efficiently via frontal instruction or digital modules, allowing practical sessions to focus on CPR skills.

Finally, this study highlights two critical but under-recognized barriers: children's fear of speaking and insufficient knowledge of their address or location. Future training should explicitly target these aspects to improve real-world emergency-call performance.

## Data Availability

The raw data supporting the conclusions of this article will be made available by the authors, without undue reservation.
